# The Role of Innate Immunity in Alcoholic Liver Disease

**DOI:** 10.35946/arcr.v37.2.08

**Published:** 2015

**Authors:** Laura E. Nagy

**Affiliations:** Laura E. Nagy, Ph.D., is a professor of molecular medicine at Case Western Reserve University School of Medicine and a staff member of the Department of Gastroenterology and Pathobiology at Cleveland Clinic Foundation, Lerner Research Institute, Cleveland, Ohio.

**Keywords:** Alcohol use, abuse and dependence, heavy alcohol drinking, alcohol effects and consequences, alcoholic liver disease, liver, gastrointestinal tract, immunity, innate immune system, immune cells, cytokines, chemokines, inflammation

## Abstract

The innate immune system represents the first-line response to invading microbes, tissue damage, or aberrant cell growth. Many of the proteins and cells involved in innate immunity are produced by, and reside in, the liver. This abundance in immune cells and proteins reflects the liver’s adaptation to various immune challenges but also makes the organ particularly vulnerable to alcohol’s effects. Heavy alcohol consumption may produce leakage of microbes and microbial products from the gastrointestinal tract, which quickly reach the liver via the portal vein. Exposure to these immune challenges and to alcohol and its breakdown products dysregulates the liver’s normally fine-tuned immune signaling pathways, leading to activation of various cellular sensors of pathogen- or damage-associated molecular patterns. The ensuing expression of pro-inflammatory cytokines (e.g., tumor necrosis factor α [TNFα], interleukin [IL]-8, and IL-1β) results in cellular dysfunction that contributes to alcoholic liver disease (ALD). Investigations into the roles of the various components of liver innate immunity in ALD have begun to uncover the molecular basis of this disease. Further progress in this area may help inform the development of interventions targeting the innate system to augment current treatments of ALD. These treatments could include antibodies against pro-inflammatory cytokines, use of anti-inflammatory cytokines, or suppression of alcohol-induced epigenetic regulators of innate immunity.

Heavy consumption of alcohol poses a well-known health risk worldwide. Alcohol’s effects on health and well-being are numerous and include injuries and fatalities resulting from alcohol-induced incapacitation. Moreover, chronic and heavy alcohol consumption affects the integrity and function of vital tissues and organs, causing slow but significant structural and functional damage over time. One of alcohol’s principal actions is damage to the liver, the primary organ for its metabolism. As a result, some 90 percent of heavy drinkers (i.e., those drinking 60 g or more of alcohol per day)—and even some who drink less— develop fatty liver (i.e., steatosis) (O’Shea et al. 2009). Up to one-third of heavy drinkers may incur more extensive liver injury, including alcoholic hepatitis, scarring (i.e., fibrosis), cirrhosis, or liver cancer (Gao et al. 2011). Moreover, about 70 percent of individuals who develop alcoholic hepatitis will progress to cirrhosis (Schwartz and Reinus 2012). The spectrum of alcohol-induced liver injuries ranging from steatosis to cirrhosis, defined here as alcoholic liver disease (ALD), is therefore a major cause of liver impairment worldwide (Gao et al. 2011).

A major contributor to ALD is alcohol-induced activation of liver innate immunity, precipitating disorders ranging from localized and transient inflammation to widespread hepatocellular injury and tissue damage (Cohen and Nagy 2011; Gao et al. 2011; Orman et al. 2013; Seki and Schnabl 2012; Wang et al. 2012). Given the pivotal role of the innate immune system in protecting the liver against foreign agents, it may seem surprising that some of the worst outcomes of alcohol-induced liver disease are the result of activation of innate immune cells. But, in fact, recent studies have revealed that alcohol induces immune activation, which drives the progression of ALD.

Innate immunity comprises chemical-physical barriers (e.g., epidermal cells, mucous membranes, and pH), as well as cellular defenses against any invading microbe or agent the immune system perceives as dangerous to the body’s cells and tissues (Gao et al. 2011). These cellular defenses, which include both immune cells (e.g., macrophages and dendritic cells) and proteins (e.g., cytokines), normally are well balanced to sense and respond to harmful agents while avoiding unnecessary immune activation. Alcohol disrupts this balance, triggering immune responses that result in inflammation (Gao et al. 2011; Seki and Schnabl 2012; Szabo et al. 2011; Wang et al. 2012). Continued high alcohol intake fuels a multistage process in which alcohol-induced liver damage advances along a continuum of steatosis, inflammation, and fibrosis, to the final stage, cirrhosis, marked by widespread tissue deformation and damage (Gao et al. 2011; Orman et al. 2013; Seki and Schnabl 2012; Wang et al. 2012).

It has been known for some time that alcohol consumption triggers inflammation of the liver, but how alcohol brings about this disease state has long remained unclear. More recently, researchers have uncovered key roles of Toll-like receptors (TLRs), whose activation during alcohol exposure results in upregulation of pro-inflammatory cytokines (e.g., tumor necrosis factor α [TNFα] and interleukin [IL]-1β) and chemokines (e.g., monocyte chemoattractant protein [MCP]-1). Moreover, these immune responses result in production of reactive oxygen species (ROS), epigenetic changes, and infiltration of tissues with circulating monocytes and neutrophils (Gao et al. 2011; Petrasek et al. 2013; Seki and Schnabl 2012; Szabo et al. 2011; Wang et al. 2012).

Although the exact molecular mechanisms through which alcohol activates innate immune cells are not entirely understood, there is increasing evidence for the close relationship between the effects of alcohol on the gastrointestinal (GI) tract and injury to the liver. Heavy alcohol consumption changes the composition of microbial communities in the GI system, tipping the balance toward more pathogenic species. Recent observations in animal models suggest that these changes are involved in promoting ALD (Yan and Schnabl 2012). Alcohol also seems to disrupt the structural integrity of the gut, causing release of bacteria and bacterial products into the circulation, which activates innate immune responses (Rao 2009; Seki and Schnabl 2012; Yan and Schnabl 2012). Because the GI tract is closely connected to the liver via the portal vein, the liver is a focal point for these alcohol-induced, gut-derived immune challenges.

Receptors located on resident immune cells in the liver (i.e., Kupffer cells; see sidebar, “Liver Cell Types and Their Roles in ALD”) sense and transmit these immune challenges. These receptors are specifically adapted to the high-challenge environment of the liver, and this adaptation contributes to the decreased responsiveness to immune challenges (i.e., liver tolerance) in healthy individuals (Petrasek et al. 2013; Seki and Schnabl 2012). However, alcohol’s effects on the gut and on immune cells, such as Kupffer cells, reduce liver tolerance and thus increase the potential for persistent inflammation. For example, microbial metabolites and cellular products released in response to the damage caused by alcohol and its metabolites activate cell surface (e.g., TLR4) and intracellular (e.g., nucleotide-binding oligomerization domain [NOD]-like) receptors (Cohen et al. 2011; Petrasek et al. 2013). This activation triggers the expression of pro-inflammatory genes, secretion of cytokines, and recruitment of various immune cells.

Additional findings suggest that alcohol exposure leads to heritable changes in how genes are expressed (e.g., epigenetic regulation) (Curtis et al. 2013). These long-lasting changes in gene expression may shift production of immune cells from anti- to pro-inflammatory cells and may induce other cellular changes that promote inflammation and ALD. Alcohol consumption also destabilizes reduction and oxidation processes (i.e., the redox balance) in the liver (Cohen et al. 2011), leading to increased production of destructive ROS that damage tissues and thus activate innate immune cells in the organ.

The goal of this review is to highlight recent advances in efforts to unravel the role of innate immunity in ALD. The following sections will focus on knowledge gleaned from recent studies of the roles of innate immune cells, proteins, and pathways in the development and progression of ALD. Although ALD is a human disease, much of the current knowledge of the role of innate immunity in ALD has been inferred from animal and in vitro cellular models of alcohol exposure. The significant degree of conservation in innate immune pathways from mouse to human bolsters the idea that many, if not most, findings in these animal and cellular models can be extrapolated to people. However, most of the information from the animal and cellular models discussed in this review awaits confirmation in studies with human subjects. The article will also explore how this knowledge may be used for treating and managing this disease.

## The Natural History of ALD

**Figure 1:**
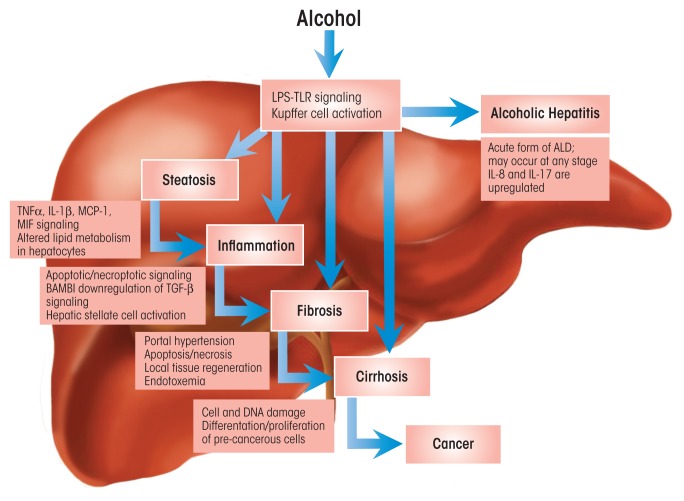
The role of innate immunity in the natural history of alcoholic liver disease (ALD). Heavy alcohol consumption causes release of bacterial products (i.e., lipopolysaccharides [LPSs]) from the gut into the bloodstream. These LPSs lead to activation of liver innate immunity by stimulating Toll-like receptor 4 (TLR 4) signaling on Kupffer cells and hepatocytes. The damaging effects of alcohol and its metabolism on cells trigger additional immune responses. Steatosis and inflammation in hepatocytes represent the early stages of ALD; continued alcohol-induced inflammation leads to apoptosis/necroptosis in hepatocytes. Downregulation of BMP and activin membrane–bound inhibitor (BAMBI) and increased transforming growth factor β (TGF-β) signaling activate hepatic stellate cells, which differentiate into myofibroblasts causing fibrosis. About 10 to 20 percent of patients with ALD (about 70 percent of patients with alcoholic hepatitis) progress to cirrhosis. Differentiation and proliferation of precancerous liver cells present in cirrhosis lead to cancer in about 10 percent of cirrhosis patients. Acute alcohol-induced inflammation (i.e., alcoholic hepatitis), characterized by high levels of pro-inflammatory cytokines (e.g., interleukin [IL]-17 and IL-8), may occur at any stage of ALD and, in severe cases, may cause death in about 50 percent of patients.

Approximately 30 percent of people who regularly consume large amounts of alcohol have a significantly increased risk for developing ALD (Lucey et al. 2009; O’Shea et al. 2010), which becomes chronic and progressively worse if alcohol consumption continues unchecked (Gao et al. 2011). The disease typically commences with the development of fatty liver (i.e., hepatic steatosis); with continued heavy alcohol consumption, steatosis may transition to inflammation, resulting in tissue damage and fibrosis (see figure 1). Ultimately, chronic ALD results in extensive organ damage and disease characterized by necrosis (i.e., cirrhosis), and in about 2 percent of cases, cancer (i.e., hepatocellular carcinoma) may develop (Orman et al. 2013; Schwartz and Reinus 2012). Alcoholic hepatitis— an acute manifestation of ALD that may coincide with clinical signs of fatty liver (in which case it is termed *alcoholic steatohepatitis*) (Lucey et al. 2009)—may occur at any stage of the disease process and significantly predisposes patients to developing cirrhosis.

The first stage in ALD, hepatic steatosis, involves several processes. Alcohol’s metabolism generates an overabundance of the metabolic intermediate nicotinamide adenine dinucleotide in its reduced form (NADH), which stimulates the synthesis of excess fatty acids in the liver (Lieber 2004). In addition, recent evidence has shown significant involvement of innate immune pathways in steatosis (Mandrekar et al. 2011). This evidence points to substantial crosstalk between metabolic and immune pathways and highlights the multifactorial nature of this initial stage. Steatosis typically resolves with abstinence from alcohol in people who have no other conditions (e.g., obesity) that promote steatosis. However, continued alcohol use may lead to alcoholic hepatitis, a moderate to severe disorder arising from acute alcohol-induced inflammation for which no highly effective treatment currently is available.

**Liver Cell Types and Their Roles in ALD**

***Kupffer Cells***

Kupffer cells are macrophages located in the liver sinusoids. They usually are among the first cells exposed to alcohol-induced, microbe-derived immunogenic challenges originating from the gut, including lipopolysaccharides (LPSs) and peptidoglycans. Kupffer cells have a dual role in mediating pro-inflammatory responses and moderating these responses through expression of anti-inflammatory cytokines. They express Toll-like receptors (TLRs), including TLR4, TLR2, TLR3, and TLR9, which, on contact with LPS, lipoteichoic acid (a component of the cell walls in Gram-positive bacteria), viral RNA, and CpG-island DNA, respectively, trigger pro-inflammatory response pathways. For example, in response to LPS stimulation, Kupffer cells produce inflammatory cytokines (e.g., tumor necrosis factor α [TNFα], interleukin [IL]-1β, IL-6, and IL-10) and several chemokines through the central regulator of inflammation, nuclear factor κB (NF-κB). Secretion of IL-12 and IL-18 activates production of interferon γ (IFN-γ) in natural killers cells, and production of transforming growth factor β 1 (TGF-β1) and ROS contribute to alcohol-induced fibrogenesis in liver tissues. Kupffer cells contribute to liver tolerance by expressing anti-inflammatory IL-10 on exposure to LPS.

***Hepatic Stellate Cells***

Hepatic stellate cells (HSCs) are activated by liver damage and express TLR2, TLR4, and TLR9, which respond to lipoteichoic acid, LPS, and CpG DNA, respectively. TLR stimulation in HSCs results in expression of IL-6, TGF-β1, and monocyte chemotactic protein (MCP-1). On activation, HSCs differentiate into myofibroblasts, representing the major producers of extracellular matrix, which contributes to fibrosis.

***Hepatocytes***

Hepatocytes are epithelial cells and the major cell type of the liver. They significantly contribute to elimination of inflammation-inducing LPS from circulating blood. LPS uptake by hepatocytes requires activity of the TLR4–CD14–MD-2 complex. Hepatocytes are the target for TNFα released by Kupffer cells in response to alcohol and LPS exposure and may undergo apoptosis or necrosis in response to TNFα receptor activation.

***Hepatic Dendritic Cells***

Activation of TLR9 and TLR7 on or in specialized plasmacytoid dendritic cells results in production of IFN-α. Conventional dendritic cells of the liver respond to LPS or lipoteichoic acid via activation of TLR4 or TLR2 by producing TNFα, IL-12, or IL-6.

***Biliary Epithelial Cells***

Biliary epithelial cells express TLRs 1 through 10 and exhibit activation of NF-κB expression and TNFα expression after stimulation with high doses of alcohol-induced LPS.

***Sinusoidal Endothelial Cells***

Sinusoidal epithelial cells (SECs) line the hepatic sinusoids and express TLR4-CD14 along with TLR9. Exposing SECs to LPS downregulates NF-κB activation, CD54 expression, and leukocyte adhesion. In these cells, LPS tolerance is not controlled via TLR4 expression, and the role of SECs in uptake of LPS in the liver is unclear.

Chronic alcohol use may also lead to the development of fibrosis (Hernandez-Gea and Friedman 2011), characterized by the generation of scar tissue composed of extracellular matrix proteins, such as collagens. As in steatosis, both aberrant metabolic processes and activation of immune responses play roles in the development and progression of fibrosis. Acetaldehyde generated during the oxidative breakdown of alcohol inhibits certain immune cells (i.e., natural killer cells) that normally moderate fibrosis by inducing apoptosis in activated hepatic stellate cells (HSCs) (Hernandez-Gea and Friedman 2011; Orman et al. 2013). In addition, cytokines secreted by Kupffer cells, as well as inflammatory scar–associated macrophages recruited from the periphery (Ramachandran and Iredale 2012), activate quiescent HSCs, resulting in the development and proliferation of extracellular matrix–producing myofibroblasts, whose activity precipitates fibrosis.

About 10 to 20 percent of patients with fibrosis who continue to heavily consume alcohol progress to the final stage of ALD, cirrhosis (Orman et al. 2013). This disease stage is characterized by widespread damage to the liver, including fibrotic deformation of tissues and blood vessels, as well as necrosis of cells. The main features of cirrhosis are the formation of nodules of varying sizes, which signify localized regeneration of lost tissues, and the obstruction of blood vessels, which causes portal hypertension. Release of immunogenic cellular debris from necrotic liver cells and the loss of the liver’s ability to clear microbial and other pro-inflammatory metabolites from the circulation results in unremitting stimulation of innate immune pathways. As a result, cirrhosis generally is associated with a poor prognosis, with a median survival time of about 10 years. Further, liver cancer (i.e., hepatocellular carcinoma) is seen in about 2 percent of patients with cirrhosis (Orman et al. 2013).

## Innate Immunity and ALD

As mentioned above, various innate immune cells and their actions play prominent and complex roles in the initiation and progression of ALD. The oxidative breakdown of alcohol by dedicated alcohol dehydrogenases and by cytochrome P450 monooxygenases generates ROS that may damage proteins, lipids, and other cellular structures. In addition, alcohol’s breakdown metabolite, acetaldehyde, exerts toxic effects on cellular structures and DNA (Wang et al. 2012). The ROS- and acetaldehyde-induced cell damage activates innate immune cells, triggering an inflammatory reaction even in the absence of invading pathogens (i.e., sterile inflammation) (Kubes and Mehal 2012). Sterile inflammation results from activation of pro-inflammatory pathways in immune and other cells carrying receptors for detecting damage-associated molecular patterns (DAMPs; also called alarmins). These molecules are released by stressed or necrotic cells, such as hepatocytes damaged by alcohol or its breakdown products. These immune pathways are essential for clearing damaged cells and cellular debris from tissues; however, their persistent activation by alcohol leads to repeated cycles of cell damage and resultant stimulation of innate immune cells, causing chronic inflammation of the liver.

Chronic alcohol intake also has more indirect effects that play a major role in ALD. Excessive alcohol consumption changes the composition of microbes found in the gut (i.e., the gut microbiome) and seems to contribute to loss of tight cellular connections in the small intestine (Rao 2009). This alcohol-induced breach of the gut barrier causes release of immunogenic compounds, primarily bacterial lipopolysaccharide (LPS, also known as endotoxin) (Bode et al. 1987) and other cell-wall constituents like peptidoglycans and microbial DNA, into the circulation. LPS is a major trigger of pro-inflammatory pathways, and its role in inflammation of the liver and stimulation of innate immunity is well established (Petrasek et al. 2013; Rao 2009; Seki and Schnabl 2012). Unmethylated CpG–containing DNAs released from bacterial cells have also emerged as a significant activator of liver innate immunity (Petrasek et al. 2013; Seki and Schnabl 2012).

In addition, alcohol depletes the levels of *S*-adenosylmethionine (SAM) (Lieber 2000) a universal methyl donor important for epigenetic regulation of transcription. Intragastric feeding of SAM in rats diminished the activity of alcohol-activated innate immune pathways (Oliva et al. 2011), highlighting the potential role of SAM in moderating innate immune responses.

### Effects on Kupffer Cells and the Complement System

**Figure 2:**
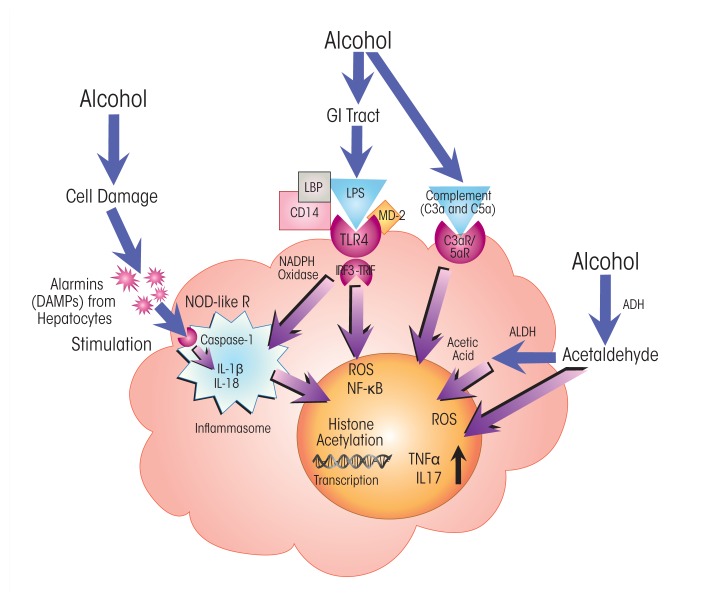
Alcohol’s effects on pro-inflammatory pathways in liver macrophages (i.e., Kupffer cells). Excessive alcohol consumption increases the permeability of the gastrointestinal (GI) tract, exposing Kupffer cells to bacterial endotoxin (i.e., lipopolysaccharide [LPS]). LPS is bound by LPS-binding protein (LBP), enabling engagement with Toll-like receptor 4 (TLR 4) and activating the myeloid differentiation primary response (MyD) 88–independent signaling pathway involving interferon regulatory factor 3 (IRF3) and TIR domain–containing adapter-inducing interferon-β (TRIF). IRF3–TRIF signaling induces production of reactive oxygen species (ROS) by nicotinamide adenine dinucleotide phosphate (NADPH) oxidases and activates nuclear factor κB (NF-κB) and histone acetylation, which trigger transcription of genes for several pro-inflammatory cytokines (i.e., tumor necrosis factor α [TNFα] and interleukin [IL]-17). Alcohol’s breakdown to acetaldehyde and acetate also stimulates ROS signaling and cytokine production. In addition, IRF3–TRIF signaling and detection of damage-associated molecular patterns (DAMPs or alarmins) released from hepatocytes after alcohol exposure stimulate the inflammasome, a multiprotein complex containing caspase 1, which cleaves and thus activates another pro-inflammatory cytokine, IL-1β. Alcohol activates complement, generating anaphylatoxins C3a and C5a, which dock with their cognate receptor on Kupffer cells, further stimulating cytokine production. NOTES: ADH = alcohol dehydrogenase; ALDH = acetaldehyde dehydrogenase; C3a/C5a R = C3a/C5a receptor; CD14 = cluster of differentiation 14 protein; MD-2 = myeloid differentiation 2 protein; NOD-like R = nucleotide-binding oligomerization domain–like receptor.

**Figure 3:**
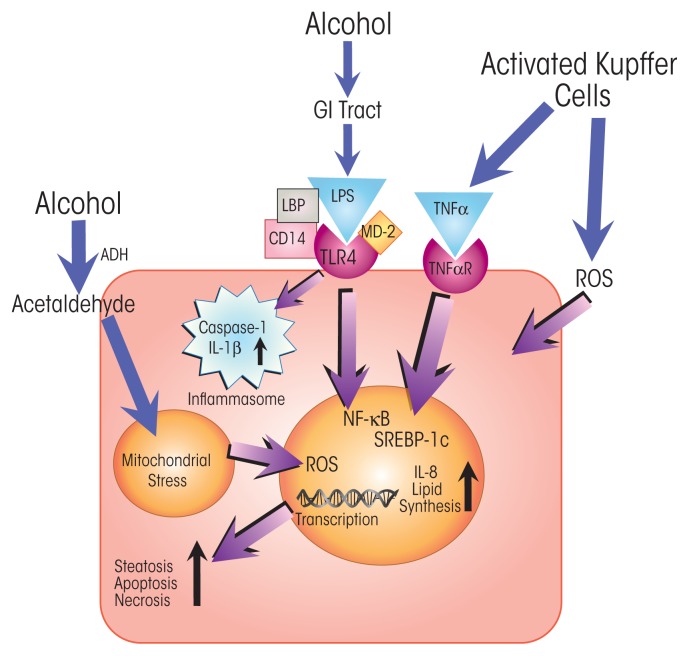
Alcohol’s direct effects on activity and viability of parenchymal liver cells (i.e., hepatocytes) and on immune-cell signaling to hepatocytes. Excessive alcohol consumption increases the permeability of the gastrointestinal (GI) tract, exposing hepatocytes to bacterial endotoxin (i.e., lipopolysaccharide [LPS]). LPS is bound by LPS-binding protein (LBP), enabling engagement with Toll-like receptor 4 (TLR4) and activation of pro-inflammatory signaling pathways. TLR4 signaling activates expression of nuclear factor κB (NF-κB), which, along with reactive oxygen species (ROS) generated in mitochondria (as a result of exposure to the toxic alcohol-breakdown product acetaldehyde) and Kupffer cells, activates transcription of pro-inflammatory cytokines (i.e., IL-8). Tumor necrosis factor α (TNFα) produced by activated Kupffer cells stimulates sterol regulatory element–binding protein 1c (SREBP-1c), which triggers expression of genes in lipid synthesis, in turn initiating the development of abnormal fat deposition (i.e., steatosis). The combined action of lipid synthesis and upregulated expression of pro-inflammatory cytokines may spur programmed cell death (i.e., apoptosis) and necrosis, resulting in alcohol-induced loss of hepatocytes from tissues NOTES: ADH = alcohol dehydrogenase; CD14 = cluster of differentiation 14 protein; MD-2 = myeloid differentiation 2 protein; TNFαR = TNFα receptor.

Kupffer cells are the resident macrophages of the liver and have key functions in innate immunity. Because of the crucial role Kupffer cells play in defending the liver against pathogens, they are among the first immune cells to respond to alcohol-induced surges of microbial metabolites, such as LPS. These bacterial products engage with the TLR4 receptors on the Kupffer cells. LPS-induced TLR4 activation stimulates the production of cytokines, including TNFα, IL-6, and IL-1β, and of chemokines, such as KC (CXCL1), MIP-2 (CXCL2), MCP-1 (CCL2), and RANTES (Gao et al. 2011; Mandrekar and Szabo 2009; Petrasek et al. 2013; Szabo et al. 2011). Secretion of these molecules from Kupffer cells, in turn, activates a pro-inflammatory cascade affecting processes in other liver cells. For example, tumor necrosis factor α (TNFα) secreted by activated Kupffer cells interacts with TNFα receptors on hepatocytes (see figures 2 and 3). TNFα receptor activation, in turn, contributes to steatosis and, ultimately, to necrosis and apoptosis of the hepatocytes that normally clear LPS and other xenobiotic compounds from the liver (Gao et al. 2011). Alcohol also affects macrophage plasticity—the environmentally determined activation to either classical pro-inflammatory (i.e., M1) or alternative anti-inflammatory (i.e., M2) macrophages. Alcohol represses activation to the M2 phenotype and thus skews macrophage distributions toward the pro-inflammatory M1 state (Louvet et al. 2011; Mandal et al. 2011).

**Figure 4:**
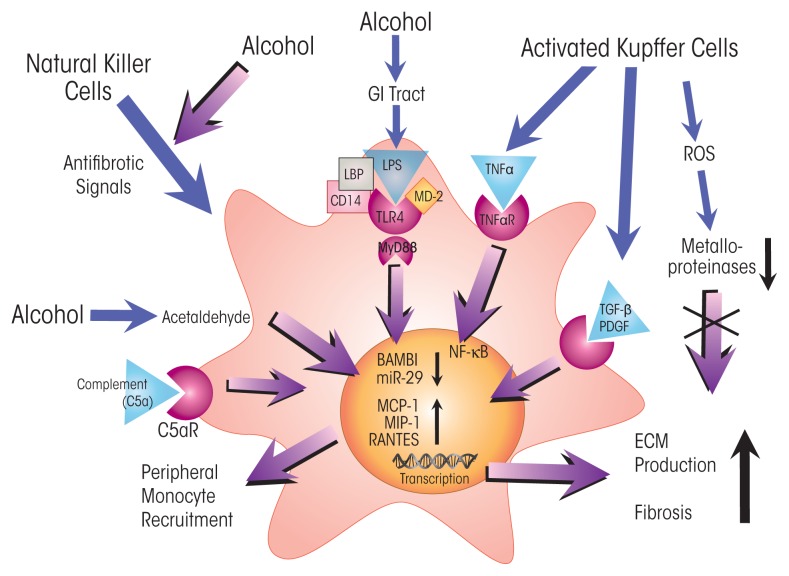
Alcohol’s effects on fibrogenic pathways in hepatic stellate cells (HSCs). HSCs are quiescent liver cells that, on stimulation by pro-inflammatory proteins and other agents, differentiate into myofibroblasts to repair damaged tissues. Excessive alcohol consumption increases the permeability of the gastrointestinal (GI) tract, exposing HSCs to endotoxin (i.e., lipopolysaccharide [LPS]). LPS is bound by LPS-binding protein (LBP), enabling engagement with Toll-like receptor 4 (TLR 4) and activating the myeloid differentiation primary response (MyD88)-dependent pathway. MyD88 signaling decreases expression of BMP and activin membrane–bound inhibitor (BAMBI), a pseudoreceptor that suppresses responses to transforming growth factor β (TGF-β; secreted by activated Kupffer cells). Thus, alcohol-induced TLR4–MyD88 signaling increases the HSCs’ responsiveness to TGF-β. microRNA 29 (miR-29) inhibits the production of extracellular matrix (ECM), and its downregulation by MyD88 signaling therefore increases ECM deposition. TLR4–MyD88 signaling in HSCs—along with complement 5a and exposure to the alcohol-breakdown product acetaldehyde and platelet-derived growth factor (PDGF) and tumor necrosis factor α (TNFα) secreted from activated Kupffer cells—upregulates the expression of various chemokines (i.e., monocyte chemotactic protein [MCP-1], macrophage inflammatory protein 1 [MIP-1], and regulated on activation, normal T cell expressed and secreted [RANTES]). These chemokines recruit macrophages (i.e., Kupffer cells and scar-associated macrophages) and other immune cells to the site where HSCs reside (i.e., the liver perisinusoidal space or space of Disse). These signals spur the differentiation of HSCs into myelofibroblasts that produce and secrete ECM, leading to liver fibrosis. In addition, Kupffer cell–produced ROS inhibit activities of metalloproteinases, which normally degrade ECM and thus inhibit fibrosis. NOTES: C5aR = C5a receptor; CD14 = cluster of differentiation 14 protein; MD-2 = myeloid differentiation 2 protein; TNFαR = TNFα receptor.

The complement system is a major antimicrobial defense pathway that straddles both innate and adaptive immunity. Most of the proteins in this system are produced in the liver. Alcohol activates complement pathways (Cohen et al. 2010) and, in heavy drinkers, can also compromise complement action by impairing liver function. Complement activation results in the production of C3a and C5a anaphylatoxins—short peptides of the complement system. Through interactions with C3a and C5a receptors, these anaphylatoxins trigger the production of pro-inflammatory innate immune proteins, such as cytokines, in leukocytes and thus contribute to inflammation (Cohen et al. 2011) (see figures 2 and 4).

### Expression and Activation of TLRs

TLRs are expressed on many cells of the liver, including Kupffer cells, endothelial cells, dendritic cells, biliary epithelial cells, HSCs, and hepatocytes. The expression of the TLRs and their level of responsiveness on these different cells normally are adjusted to promote appropriate reactions to immune challenges and prevent misplaced and potentially damaging responses (Petrasek et al. 2013). Alcohol exposure turns up the dial of this finely tuned TLR network, heightening TLR responses to external and internal triggers, such as pathogen-associated molecular patterns (PAMPs) and DAMPs, respectively (Petrasek et al. 2013; Seki and Schnabl 2012).

TLR4 plays a very prominent role in alcohol-induced inflammation, activating two distinct signaling pathways—the myeloid differentiation primary response gene 88 (MyD88)-dependent pathway and the MyD88-independent pathway (Petrasek et al. 2013; Seki and Schnabl 2012; Wang et al. 2012). Engagement of the MyD88-dependent TLR pathway triggers expression of nuclear factor kappa B (NF-κB), a central transcriptional regulator of immune responses and pro-inflammatory pathways. TLR4-mediated, MyD88-dependent signaling also promotes mitogen-activated protein kinase (MAPK)-induced production of cytokines, including TNFα. The MyD88-independent pathway proceeds via a different major adaptor protein, TIR domain–containing adapter-inducing interferon-β (TRIF), and results in production of interferon regulatory factor 3 (IRF3), type 1 interferons (IFNs), and pro-inflammatory cytokines.

Experiments in rodent models of ALD have demonstrated that TLR4–TRIF signaling plays an essential role in alcohol-induced activation of TLR4 in Kupffer cells (Hritz et al. 2008; Mandal et al. 2010*b*) (see figure 2). Moreover, TLR4 signaling in both immune cells (i.e., Kupffer cells) and nonimmune cells involved in tissue repair (i.e., HSCs) is required for the development of alcoholic hepatitis and fibrosis (Inokuchi et al. 2011). Co-receptor proteins (e.g., cluster of differentiation [CD] 14 and myeloid differentiation [MD] 2) influence the responsiveness of TLR4 to receptor ligands, such as LPS.

### Cytokines and Chemokines in ALD

Cytokines are relatively small proteins (i.e., less than 30 kDa in size), many of which are produced by various cells in response to injury or contact with pathogens. Patients with ALD often have elevated levels of various cytokines (see sidebar, “Key Cytokines and Hormonal Peptides in ALD”) (Szabo et al. 2011). The cytokines are key components of the innate immune system, facilitating cell-to-cell communication and regulating proliferation and maturation of cell populations in response to immune challenges and environmental changes. Cytokines engage with cells via specific receptors on the cellular surfaces, sometimes triggering their own (i.e., autocrine) production in the cells or amplifying or inhibiting the activities of other cytokines. These interactive cytokine networks play an indispensable role in mediating innate immune responses, and their relative contributions define the different types and outcomes of these responses. A subset of cytokines, the chemokines, recruit immune cells, such as neutrophils and lymphocytes, to sites of injury or infection; thus, chemokines are often involved in pro-inflammatory signaling pathways.

As can be expected from alcohol’s effect on the innate immune cells of the liver, such as Kupffer cells, and on LPS-activated TLR signaling, heavy alcohol consumption stimulates the production of many cytokines. One of the major cytokines in ALD and one of the first to be associated with the condition is TNFα (McClain and Cohen 1989). TNFα is expressed early in response to alcohol exposure, and its production coincides with liver damage; moreover, abolishing its expression in animal models of ALD mitigates liver injury (Gao 2012; Wang et al. 2012). These observations underscore that TNFα’s prominent pro-inflammatory role in ALD and its activity significantly contributes to alcohol-induced liver damage.

Interleukins are also among the pro-inflammatory cytokines implicated in ALD. For example, TNFα, along with other NF-κB–induced agents, stimulates the expression of IL-8, whose levels are greatly increased in alcoholic hepatitis (Sheron et al. 1993). IL-17 is also upregulated in ALD, and although its activity is lower than that of TNFα, it seems to play a role in both inflammation and fibrosis of the liver (Lemmers et al. 2009).

Alcohol exposure also induces expression of anti-inflammatory cytokines. For example, three interleukins— IL-6, IL-10, and IL-22—activate signal transducer and activator of transcription 3 (STAT3), a transcriptional regulator of an array of genes involved in immunity and cellular defenses and differentiation (Gao 2012; Wang et al. 2012). IL-22 binds to specific receptors on epithelial cells and on hepatocytes and triggers the expression of anti-apoptotic and anti-oxidative stress genes while repressing genes involved in lipid production (Gao 2012; Wang et al. 2012). It often remains unclear whether the expression of anti-inflammatory cytokines reflects a compensatory response of the immune system to the alcohol-induced upregulation of the pro-inflammatory cytokines or to the cell damage alcohol produces.

Interestingly, although IL-6, IL-10, and IL-22 all stimulate STAT3, only IL-22 seems to have solely anti-inflammatory effects protecting against acute and chronic liver damage (Park et al. 2011). IL-6 and IL-10, in contrast, have dual roles as both pro- and anti-inflammatory proteins in ALD (Gao 2012; Wang et al. 2012). Their specific effects seem to be determined by the cell type affected and the stage of ALD. For example, IL-6 increases the expression of pro-inflammatory cytokines in Kupffer cells (Gao 2012), but its activity also protects hepatocytes. Recent findings suggest that alcohol-induced oxidative stress stimulates the expression of IL-6, promoting senescence in hepatocytes, which, in turn, makes cells more resistant to steatosis and apoptosis (Wan et al. 2014). IL-10 blocks the activation of TNFα and complement, thus reducing expression of pro-inflammatory pathways; but it also checks expression of IL-6, thus limiting liver regeneration afforded by IL-6–induced upregulation of expression of liver-protective genes (Gao 2012).

Chemokines also play critical roles in alcohol-induced inflammation. For example, the levels of MCP-1 (also known as CCL2) are elevated in patients with ALD, and upregulated MCP-1 expression is also observed in Kupffer cells and hepatocytes of alcohol-fed mice (Mandrekar et al. 2011). Feeding alcohol to MCP-1–deficient mice results in less steatosis, lower expression of pro-inflammatory cytokines (i.e., of TNFα, IL-1β, and IL-6), and lower levels of oxidative stress than in wild-type mice (Mandrekar et al. 2011). Moreover, MCP-1 is required for activating cytokine expression in response to LPS (Mandrekar et al. 2011). In patients with ALD, increased MCP-1 expression is associated with increased disease severity and elevated levels of the pro-inflammatory cytokine IL-8 (Dégre et al. 2012). One major role of MCP-1 in ALD is to recruit neutrophils to inflamed liver tissues. However, because circulating neutrophils in the ALD patients lack MCP-1 receptors (Dégre et al. 2012), the exact mechanisms by which MCP-1 exerts its control over neutrophil movement remain to be elucidated. Nevertheless, these results connect chemokines to lipid metabolism in the liver and suggest that MCP-1 plays a major role in alcohol-induced liver inflammation by activating several pro-inflammatory cytokines in response to common triggers of ALD and by promoting neutrophil infiltration into liver tissues.

Barnes and colleagues (2013) recently demonstrated that macrophage migration inhibitory factor (MIF)—a multifunctional pro-inflammatory cytokine and chemokine, which also has some hormonal features—has a critical role in both the early and chronic stages of liver injury in a mouse model of ALD. MIF-deficient mice are protected against several of alcohol’s effects on the innate immune system, including inflammation, and also against hepatocyte damage and apoptosis. Similar to MCP-1, MIF plays a role in alcohol-induced lipid accumulation in liver cells (Barnes et al. 2013), suggesting a role for both chemokines in regulating lipid metabolism directly or indirectly. The findings lend further support to existing evidence that links innate immune pathways and proteins to the regulation of fundamental metabolic processes.

**Key Cytokines and Hormonal Peptides in ALD**

***Tumor Necrosis Factor*** α

Tumor necrosis factor α (TNFα) is a major pro-inflammatory cytokine whose levels are increased in the blood and liver of individuals with alcoholic liver disease (ALD). TNFα expression is regulated by the transcription factor nuclear factor kappa B (NF-κB). It is upregulated in macrophages (i.e., Kupffer cells) as well as in circulating monocytes in response to Toll-like receptor 4 (TLR4) activation by bacterial endotoxin (i.e., lipopolysaccharide [LPS]) and by the breakdown products of alcohol, acetaldehyde and acetic acid. TNFα induces necrosis and apoptosis in hepatocytes, thus contributing to inflammation in ALD. TNFα repression by the phosphodiester-inhibitor pentoxifylline and by treatment with TNFα antibody alleviates TNFα-induced liver damage in mice and improves the short-term survival of ALD patients, respectively, but increases the risk for infections in ALD patients.

***Interleukin 1***β

Interleukin-1β (IL-1β) along with type I IL-1 receptor (IL-1R1), and IL-1 receptor antagonist (IL-1Ra), is an important regulator of the IL-1 signaling complex. This complex plays a critical role in alcohol-induced hepatic steatosis, inflammation, and damage. IL-1β activation is mediated through the inflammasome, a multiprotein complex in macrophages that senses and transduces endogenous danger signals via IL-1β cleavage by caspase-1. IL-1β increases the activity of pro-inflammatory monocyte chemotactic protein (MCP-1) in hepatocytes and contributes to increased TLR4-dependent pro-inflammatory signaling in macrophages.

***IL-6***

IL-6 has both pro- and anti-inflammatory activities. It increases expression of pro-inflammatory cytokines in macrophages and decreases necrosis-associated inflammation in hepatocytes, which aids recovery from injury and facilitates tissue regeneration. Along with IL-10 and IL-22, IL-6 activates signal transducer and activator of transcription 3 (STAT3), which controls expression of a set of genes involved in innate immunity and in cell survival and differentiation. IL-6 release from M2 macrophages induces senescence and blocks apoptosis and steatosis in hepatocytes in the early stage of alcohol-induced liver injury in mice. IL-6 activates STAT3 in sinusoidal endothelial cells of the liver, thereby increasing cell survival. IL-6 levels, along with those of IL-8 and IL-10, are increased in patients with ALD who have no clinical signs of liver disease.

***IL-8***

IL-8 is released from injured hepatocytes and has important pro-inflammatory roles as a chemokine that recruits neutrophils to sites of inflammation. Its expression is induced by TNFα via activation through NF-κB. IL-8 levels are greatly increased in people with acute alcoholic hepatitis but are only moderately upregulated in those with cirrhosis. IL-8 levels, along with those for IL-6 and IL-10, are elevated in individuals with alcoholism who have no signs of liver disease.

***IL-10***

IL-10 is a strong suppressor of inflammation by preventing production of the pro-inflammatory cytokines TNFα, IL-1β, and IL-6 in macrophages. However, its anti-inflammatory, hepatoprotective effects are contingent on the expression of other cytokines, and its inhibitory effect on IL-6 expression can delay liver regeneration and increase steatosis. IL-10 expression is moderately to highly increased in ALD and, along with that of IL-6 and IL-8, is also upregulated in alcoholic patients without signs of liver disease. IL-10 acts only on immune cells expressing its cognate receptors and facilitates sustained activation of the transcription factor STAT3 in Kupffer cells, thus inhibiting inflammation. IL-10 also inhibits fibrosis.

***IL-17***

IL-17 is a recently discovered, pro-inflammatory chemokine whose levels are increased in people with ALD. It is produced by monocytes and T cells and plays an important role in recruiting neutrophils to inflamed liver tissues. It may act in concert with TNFα to activate NF-κB, thereby inducing expression of other pro-inflammatory cytokines. IL-17’s main targets are hepatic stellate cells (HSCs), in which it induces production of pro-inflammatory IL-8. IL-17 is also thought to be involved in the development of fibrosis.

***IL-22***

IL-22 is an anti-inflammatory cytokine whose expression limits steatosis and liver damage. The IL-22 targets are hepatocytes in which it activates the transcription factor STAT3. The antioxidant, antiapoptotic, and antisteatotic actions of IL-22 make it a promising target of interventions for treating ALD with minimal side effects because of the restricted distribution of IL-22 receptors.

***Adiponectin***

Adiponectin is an adipokine, a peptide hormone, whose secretion from fat cells (i.e., adipocytes) is inhibited by alcohol. Adiponectin increases fatty acid oxidation and thus suppresses steatosis. It also decreases expression of the pro-inflammatory cytokine TNF-α in macrophages (i.e., Kupffer cells) by inducing expression of heme oxygenase 1 (HO-1), which decreases TLR4/MyD88-independent signaling, and by increasing polarization to anti-inflammatory M2 macrophages. In addition, adiponectin upregulates expression of anti-inflammatory IL-10.

The effect of the hormonal peptide adiponectin on innate immunity, specifically on anti-inflammatory cytokine production and activity, is also worth noting. Adiponectin is secreted by fat cells (i.e., adipocytes) and has been shown to alleviate steatosis, inflammation, and liver damage in animal models (Xu et al. 2003). Recent evidence suggests that adiponectin moderates alcohol-induced production of pro-inflammatory TNFα and promotes expression of IL-10 (Mandal et al. 2010*a*). Because IL-10 activates STAT3, its activation by adiponectin lowers inflammation by stimulating STAT3-induced expression of anti-inflammatory genes in myeloid cells, such as Kupffer cells. In addition, adiponectin stimulates heme oxygenase 1 (HO-1), which suppresses the pro-inflammatory TLR4-dependent/MyD88-independent pathway in Kupffer cells (Mandal et al. 2010*b*). Alcohol-induced oxidative stress decreases secretion of adiponectin by adipocytes (Tang et al. 2012), which links oxidative stress to decreased levels of liver-protective, anti-inflammatory hormones, representing yet another mechanism by which alcohol perturbs liver innate immunity.

Another small peptide, ghrelin, which is produced mainly in the gut but also in the liver, has been shown to promote antifibrotic and hepatoprotective effects in both animals and humans with hepatic fibrosis (Moreno et al. 2010). Ghrelin decreases activation of NF-κB in hepatocytes, which attenuates apoptotic signaling in these cells. It also limits expression of collagen- α1 and TGF-β1 but not of NF-κB and IL-8 in HSCs, indicating that ghrelin protects liver tissues mainly by suppressing fibrogenic activities in liver cells (Moreno et al. 2010).

### Activation of the Inflammasome and IL-1β Expression

Recent observations by Petrasek and colleagues (2012) in a mouse model of alcoholic hepatitis support significant involvement of another important cytokine, IL-1β—which has roles in rheumatoid arthritis and autoimmune disorders—in alcohol-induced inflammation. The researchers show that the IL-1β signaling complex is essential for the initiation of alcohol-induced inflammation and progression to liver fibrosis. IL-1β is activated through the inflammasome, a large protein assembly composed of NOD-like receptor proteins that sense alarmins, the caspase-1 (Casp-1) protein (an enzyme that cleaves other proteins to activate them), and an apoptosis-associated speck-like CARD-domain-containing (ASC) protein (Szabo and Csak 2012) (see figures 2 and 3). The inflammasome is activated in Kupffer cells of alcohol-fed mice (Petrasek et al. 2012) as well as in hepatocytes exposed to LPS and fatty acids (Csak et al. 2011). It is stimulated by intracellular signals such as alarmins, which engage with pattern-recognition domains of the NOD-like receptors.

Inflammasome activity in Kupffer cells promotes Casp-1–mediated cleavage, and thus activation, of IL-1β (Petrasek et al. 2012). Blocking IL-1β activity strongly decreases liver inflammation and damage (Petrasek et al. 2012). These findings further amplify the view that IL-1β acts as a pro-inflammatory cytokine in immune cells in ALD and that IL-1β signaling through the inflammasome is required for alcohol-induced liver injury. Coupled with the observations of Mandrekar and colleagues (2011) on MCP-1, these findings also establish a link between innate immune activity and steatosis. This association is further supported by the observation that steatosis can be prevented when innate immune responses are experimentally abrogated by checking IL-1β or MCP-1 activity. Additional investigations into pro-inflammatory signaling in the methyl-choline deficiency model of non-alcoholic steatohepatitis have indicated that activation of the inflammasome and generation of IL-1β are independent of TLR4 but contingent on the MyD88-dependent pathway (Csak et al. 2014).

### Cytokine Effects on Hepatocytes and HSCs

Alcohol-induced increases in LPS stimulate TNFα production and its release primarily from Kupffer cells (McClain and Cohen 1989; Wang et al. 2012). In addition, alcohol sensitizes other liver cells to TNFα’s actions (An et al. 2012). One distinct role of TNFα is to induce programmed cell-death pathways (i.e., apoptosis and necroptosis) by binding to and activating death receptors on cells. Activation of these receptors results in the expression of pro-apoptotic or necroptotic mediators (e.g., caspases and receptor-interacting proteins). Alcohol thus has profound effects on cell viability by activating the expression of cytotoxic cytokines and increasing cell sensitivity to the actions of these cytokines. For instance, whereas hepatocytes in healthy people are not highly sensitive to TNFα activation of death receptor pathways, alcohol primes cells for the TNFα-mediated stimulation of cell-death pathways (Pastorino and Hoek 2000).

TNFα binds to and activates two receptors—TNF-R1 and TNF-R2—and the outcome of their activation hinges on the relative levels of stimulation of three main pathways: a pro-apoptotic pathway, a pro-necroptotic pathway, and a cell-survival pathway (Vanden Berghe et al. 2014). Binding to TNF-R1 activates apoptotic and necrotic pathways through a signaling cascade involving multiple proteins, including TNF-R1–associated death domain protein (TRADD); TNF receptor–associated factor 2 (TRAF2); and caspases 8, 3, and 7. TNF-R1 activation also may result in the stimulation of a cell-survival pathway involving NF-κB. In contrast, activation of TNF-R2 stimulates only cell survival (Malhi et al. 2010).

Apoptosis is an important regulatory mechanism for controlling the size of cell populations (e.g., neutrophils) or to avoid unchecked proliferation of abnormal cells. However, apoptosis triggered via the action of alcohol-induced TNFα on, for example, hepatocytes can severely impair liver function. Recent evidence also indicates that chronic ethanol feeding can activate necroptotic cell-death pathways in hepatocytes. Mice in which an important necroptotic regulator—receptor-interacting protein kinase (RIP)-3—is inactivated are protected from alcohol-induced liver injury (Roychowdhury et al. 2013). Hepatocytes are the major cell type in the liver and, along with Kupffer cells, eliminate most of the LPS from circulation. Widespread apoptosis and necrosis of hepatocytes therefore increase levels of circulating LPS, which in turn fuels further inflammation through activation of TLR4 on Kupffer cells and ultimately escalates the release of pro-apoptotic TNFα. This helps explain why patients with severe liver disease (i.e., cirrhosis) often show high levels of LPS in the blood (i.e., endotoxemia), resulting in sepsis (Bode et al. 1987).

Persistent alcohol-induced activation of cytokines in immune cells such as Kupffer cells also promotes fibrosis. Production and secretion of transforming growth factor (TGF)-β and of platelet-derived growth factor (PDGF) from Kupffer cells or from inflammatory scar–associated macrophages activates HSCs (see figure 4), triggering them to develop into myofibroblasts. Moreover, HSCs may be further activated by engulfing apoptotic hepatocytes. HSCs express a number of receptors involved in innate immunity, including the C5a receptor, whose ligand, C5a, is a potent mitogen that may stimulate HSC migration (Das et al. 2014). HSCs also express TLR4, which senses LPS. Although LPS alone cannot activate HSCs, its action via TLR4 downregulates expression of BMP and activin membrane-bound inhibitor (BAMBI), thereby sensitizing the cells to activation by TGF-β (Liu et al. 2014; Seki et al. 2007). BAMBI is a pseudoreceptor, which in quiescent HSCs diminishes TGF-β’s activity by binding to it without triggering intracellular TGF-β signaling. As shown by Seki and colleagues (2007), decreased BAMBI expression sensitizes HSCs to TGF-β activation, and LPS-induced TLR4 activation stimulates chemokine secretion along with recruitment and activation of Kupffer cells. Moreover, LPS activates TLR4 signaling in HSCs through the MyD88-dependent NF-κB pathway, demonstrating involvement of this key innate immune pathway in fibrosis and recruitment of immune cells. NF-κB directly binds the BAMBI promoter, along with histone deacetylase (HDAC) 1, thus repressing BAMBI expression and promoting TGF-β activation (Liu et al. 2014).

Activated HSCs produce and secrete extracellular matrix proteins (i.e., collagens), resulting in fibrogenesis (Hernandez-Gea and Friedman 2011; Seki and Schnabl 2012). Although fibrogenesis is essential for normal tissue repair, its dysregulation by recurrent activation of cytokines acting on HSCs precipitates inflammatory fibrosis. This stage of ALD involves the formation of scar tissue, which interferes with normal tissue function and often results in portal hypertension (Hernandez-Gea and Friedman 2011). Another cytokine pathway involving HSCs and leading to fibrosis in ALD patients involves IL-17. This cytokine, which is produced by peripheral blood cells (i.e., Th17 lymphocytes) in patients with alcoholic hepatitis and cirrhosis, stimulates its cognate receptors on HSCs (Lemmers et al. 2009). In response, the cells secrete IL-8 and growth-related oncogene (GRO)-α, recruiting neutrophils to their tissue, resulting in localized pro-inflammatory immune-cell infiltrates and fibrosis scores closely correlated with IL-17 levels.

Together, these findings indicate that cytokines produced by immune cells, such as TNFα produced by Kupffer cells and IL-17 produced by Th-17 lymphocytes, play a major role in ALD by affecting the viability and function of hepatocytes and by activating quiescent HSCs to produce excess extracellular proteins. These observations thus provide critical insight into the molecular processes and mechanisms that produce liver damage and fibrosis in ALD.

## Epigenetic Effects

Alcohol exerts additional effects on the innate immune system, for example, by producing epigenetic changes in the expression of genes for pro- and anti-inflammatory pathways (Curtis et al. 2013). Epigenetic changes affect the activity at gene promoters or entire gene regions and can have long-term and even heritable effects on gene expression without altering the underlying DNA sequence. Three main mechanisms operate in epigenetics:

- DNA methyltransferases, using SAM as methyl donor, methylate a cytosine nucleotide at CpG-rich regions (i.e., CpG islands) in the DNA of gene promoters, which decreases expression of the downstream genes.
- Methylation, acetylation, phosphorylation, ubiquitination, or sumoylation of the proteins around which DNA is coiled (i.e., histones) alters accessibility of the transcriptional proteins to the DNA.
- Some investigators are now extending the concept of epigenetics to include transcriptional regulation by microRNAs (miRNAs). These molecules regulate the expression of mRNAs with which they share similar sequences (Curtis et al. 2013).

The study of alcohol’s effects on epigenetic regulation and of the mechanisms by which alcohol exerts these effects has been a rapidly emerging field over the past decade. Insight gleaned from initial studies has shown that alcohol can interfere with the fundamental processes of epigenetic regulation in people with ALD as well as in animal models of the disease or in cultured human cells exposed to alcohol or its metabolic byproducts (reviewed by Kruman and Fowler 2014; Mandrekar 2011; Shukla and Lim 2013).

Studies in animals and in human cells lines have demonstrated that alcohol and LPS increase the expression of microRNA-34a, which helps alleviate alcohol-induced apoptosis in hepatocytes and biliary epithelial cells by targeting caspase 2 and sirtuin 1. The elevated expression of miRNA-34a is the result of an alcohol-induced decrease in methylation (i.e., hypomethylation) at a CpG island in the miRNA-34a promoter (Meng et al. 2012).

Alcohol also alters the cellular levels of SAM and of histone acetyltransferases (HATs) and deacetylases (HDACs), whose activities make DNA more or less accessible, respectively, to gene transcription. For example, the histone deacetylase HDAC1 has been shown to play a critical role in the silenced expression in HSCs of the fibrosis-attenuating protein BAMBI (see figure 4) (Liu et al. 2014). In addition, chemical inhibition of HDAC activity seems to reduce inflammation by reversing an alcohol-induced perturbation in macrophage polarization that results in a greater proportion of pro-inflammatory (M1) macrophages (Curtis et al. 2013). Oxidative stress caused by alcohol metabolism also triggers epigenetic changes, and alcohol-induced release of LPS and activation of TLR4 affects both HAT and HDAC activities, resulting in epigenetic changes in DNA regions containing genes for pro-inflammatory cytokines (Curtis et al. 2013).

## Approaches for Resolving Alcohol-Induced Liver Inflammation

Standard interventions for treating ALD depend on the stage and severity of the disease and typically include counseling abstinence from alcohol use; administration of corticosteroids (to inhibit alcohol-induced, pro-inflammatory pathways) and nutritional support for alcoholic hepatitis; and, in advanced cases, liver transplantation (Gao and Bataller 2011; Orman et al. 2013). Although rates of disability and death caused by ALD remain high despite these interventions, such treatments can significantly improve quality of life and avert early death caused by ALD. Overturning earlier assumptions about the persistence of alcohol-induced liver damage, recent studies have reported that some of the tissue injuries present even in the advanced stages of ALD, such as fibrosis, are reversible (Hernandez-Gea and Friedman 2011). This makes the discovery of new treatments that can augment existing ones even more urgent.

Recognition of the central role of innate immunity in ALD has spurred research into modulating the activity of key immune cells and cytokines. To this end, the discovery of the central role of TNFα in promoting inflammation in ALD prompted studies in which antibodies against TNFα were used to alleviate alcohol-induced inflammation. TNFα antibodies indeed significantly dampen liver inflammation (Gao 2012), but because TNFα is critical to fighting microbial pathogens, this approach often increases the risk for serious infections in ALD patients.

The limitations of the above approach highlight that cytokine-based interventions will need to be carefully calibrated according to the activity profile and radius of action of each cytokine to minimize or prevent adverse effects. For instance, exploitation of the hepatoprotective properties of IL-6 is limited by the abundance of IL-6 receptors in many tissues, potentially resulting in off-target effects. However, as proposed by Gao (2012), using IL-6 in ex vivo treatment of donor livers to reverse minor organ damage (e.g., steatosis) before transplantation into ALD patients could have some utility.

Current approaches for interventions in vivo focus on those immune regulators that target only a few cells or tissues. As discussed previously, IL-22 has an array of hepatoprotective activities, including antioxidant, antimicrobial, and antiapoptotic effects. Moreover, expression of the IL-22 receptor, IL-22R, is confined to epithelial cells, such as hepatocytes. This has led to the proposition that combining the use of IL-22 with anti-inflammatory corticosteroids and TNFα inhibitors could offset the immunosuppressing effects of these two agents and promote recovery of liver tissues (Gao 2012). However, because evidence from animal models suggests that IL-22 may play a role in the development of hepatic carcinoma (Park et al. 2011), such use would be restricted to ALD patients who do not have cirrhosis (which may contain precancerous cells) or liver cancer (Gao 2012).

The discovery of the role of epigenetic factors in the development of ALD and their effects on immune cells and responses opens the way to possible interventions that target key epigenetic regulators and processes in ALD. For example, because HDAC1 seems to make HSCs more receptive to the fibrosis-inducing action of TGFβ (Liu et al. 2014), inactivation of HDAC1 via antibodies or chemical agents may augment current treatments for halting or reversing fibrosis in patients with ALD. In addition, chemical inactivation of HDACs involved in alcohol’s effects on macrophage polarization to pro-inflammatory M1 macrophages may help reduce inflammation, steatosis, and fibrosis in tissues. Thus, HDAC inhibitors or activators of HATs that prevent or reverse the effects of HDACs may someday prove useful in the treatment of ALD.

Finally, technological advances to sequence and analyze DNA of patients has helped identify key genetic variants such as single-nucleotide polymorphisms (SNPs) in genes involved in liver diseases (Guo et al. 2009; Singal et al. 2014). Although in its early stages and not yet fully extended to the specific etiology of ALD, genetic profiling of ALD patients for SNP variants in genes involved in innate immune pathways could help identify patients vulnerable to advanced stages of ALD (e.g., cirrhosis) (Guo et al. 2009). Such personalized-medicine approaches could significantly improve the success and cost-effectiveness of current treatments and spur development of new interventions for ALD.

## Conclusions

Innate immunity plays a central role in ALD, and recent studies have uncovered several pivotal molecular mechanisms underlying alcohol’s effects on the immune system of the liver. Excessive consumption of alcohol alters the characteristics and composition of the microbiome in the GI tract and increases translocation of bacteria and bacterial products, such as LPS and peptidoglycans, from the gut via the portal system to the liver. This increased influx of LPS, along with the direct effects of alcohol on immune cells and liver tissues, activates innate immune pathways. This activation occurs via stimulation of TLRs and through sensors of cell damage on or in the immune cells of the liver, such as Kupffer cells. These processes lead to the production of several pro-inflammatory cytokines (e.g., of TNFα, IL-1β, IL-8, and IL-17), triggering steatosis in hepatocytes and inducing fibrogenic pathways in HSCs. Moreover, production of chemokines, such as MCP-1 and MIF, leads to the infiltration of liver tissues by monocytes, neutrophils, and dendritic cells whose activities can further increase inflammation and impede recovery. Alcohol and its metabolic breakdown products acetaldehyde and acetate, along with ROS produced during alcohol metabolism, generate oxidative stress and affect epigenetic regulation that trigger activation of pro-inflammatory pathways such as polarization to M1 macrophages.

The insight gleaned from these complex interactions and pathways may provide the impetus for devising treatments using pro- or anti-inflammatory cytokines that act on defined cell types or employing agents that control epigenetic regulators to expand currently available interventions for treating ALD.
